# Regulation of CeA-Vme projection in masseter hyperactivity caused by restraint stress

**DOI:** 10.3389/fncel.2024.1509020

**Published:** 2024-11-21

**Authors:** Ya-Juan Zhao, Ji Chen, Yang Liu, Lv-La Pan, Yan-Xia Guo, Zhou-Ming Zhang, Qiang Li, Yong-Jin Chen

**Affiliations:** ^1^State Key Laboratory of Oral and Maxillofacial Reconstruction and Regneration, Department of General Dentistry and Emergency, National Clinical Research Center for Oral Diseases, Shaanxi International Joint Research Center for Oral Diseases, School of Stomatology, The Fourth Military Medical University, Xi’an, China; ^2^State Key Laboratory of Oral and Maxillofacial Reconstruction and Regeneration, Department of Oral Implantology, National Clinical Research Center for Oral Diseases, Shaanxi Engineering Research Center for Dental Materials and Advanced Manufacture, School of Stomatology, The Fourth Military Medical University, Xi’an, China; ^3^Department of Anatomy, Basic Medical College, Zhengzhou University, Zhengzhou, China

**Keywords:** central amygdala, mesencephalic trigeminal nucleus, restraint stress, anxiety, masticatory muscle, overactivity

## Abstract

The overactivity of the masticatory muscles (bruxism or teeth clenching) is associated with stress exposure, and often leading to consistent muscle pain. However, the neural mechanism underlining it is not fully understood. The central amygdala (CeA), which is linked to stress-induced behaviors and physical reactions, projects directly to the mesencephalic trigeminal nucleus (Vme), which is crucial for oral–motor coordination. Thus, we hypothesized that the projections from the CeA to the Vme could be linked to stress-induced anxiety and overactivity of the jaw muscles. After establishing an animal model of restraint stress, we found that chronic stress could lead to noticeable anxiety-related behavior, increased masseter muscle activity, activation of GABAergic neurons in the CeA, and opposite changes in the excitability of multipolar GABAergic interneurons and pseudounipolar excitatory neurons in the Vme. Subsequently, through the utilization of anterograde and transsynaptic tracing in conjunction with immunofluorescence staining, we discovered that the neural projections from the CeA to the Vme were mainly GABAergic and that the projections from the CeA terminated on GABAergic interneurons within the Vme. Moreover, chemogenetically suppressing the function of GABAergic neurons in the CeA could effectively reduce anxiety levels and reverse the increase in the activity of the masseter muscles induced by stress. And, specifically inhibiting GABAergic projections from the CeA to the Vme via optogenetics could reduce the hyperactivity of the masseter muscles but not stress-induced anxiety. In conclusion, our findings indicate that GABAergic projections from the CeA to the Vme may play an important role in the masseter overactivity in response to chronic stress.

## 1 Introduction

Orofacial muscle pain is the most common (up to 80%) and bothersome symptom in patients with temporomandibular disorders (TMDs) (Beaumont et al., 2020; Singh et al., 2024). Numerous studies have shown that psychological stress plays a significant role in the development of chronic orofacial pain (Weber et al., 2016; Ettlin et al., 2021). Individuals exposed to long-term stressors are more prone to developing orofacial muscle tension and fatigue, potentially leading to persistent jaw muscle pain (Janal et al., 2021; Silva et al., 2022; Solis et al., 2024). Furthermore, animals subjected to stress exhibit behaviors resembling anxiety and increased activity of and irregular energy metabolism in their jaw muscles (Miao et al., 2022; Pereira et al., 2019; Xiao et al., 2023). Whether there are neural pathways in the central nervous system that are responsible for stress-induced overactivity of the masticatory muscles remains unclear.

The central amygdala (CeA), which is part of the amygdala complex, functions as a mainly integrative hub that converts stress-related information, including fear- and anxiety-inducing information, into behavioral and physiological responses, mostly by projecting to brainstem nuclei (Gilpin et al., 2015; Weera et al., 2023; O’Leary et al., 2022). Previous studies have confirmed that the CeA projects directly to the mesencephalic trigeminal nucleus (Vme) (Lazarov et al., 2011; Shirasu et al., 2011), which controls oral–motor patterns by transmitting proprioceptive signals from the masticatory muscles and projecting to the trigeminal motor nucleus (Vmo), which regulates the activity of the masticatory muscles (Lazarov, 2007; Lipovsek et al., 2017; Liu et al., 2019). In our previous study, stress was found to increase the activity of Vme neurons, strengthen glutamatergic neural projections from the Vme to the Vmo, elevate Vmo activity, and cause excessive masseter muscle activity (Zhao et al., 2022). Consequently, whether the CeA-Vme neural pathway is one of the central pathways through which stress can is translated into oral-motor hyperactivity needs to be further explored.

Approximately 95% of neurons in the CeA contain the inhibitory neurotransmitter *γ*-aminobutyric acid (GABA) (Babaev et al., 2018), and the CeA acts as the main output region of the amygdala (Whittle et al., 2021). There are two kinds of neurons in Vme, including large pseudounipolar neurons and small multipolar neurons. The former are excitatory glutamatergic neurons and are also parvalbumin (PV) positive that relay mainly oral proprioceptive information and make synaptic connections with motor neurons in the Vmo responsible for jaw closing; the latter are usually GABAergic (inhibitory) neurons and act as interneurons to regulate local circuits (Lazarov, 2000; Lazarov, 2002). However, the specific neurons that project from the CeA to the Vme and the potential role of GABAergic interneurons from the Vme in jaw muscle overactivity induced by stress remain unclear.

Considering the above information, we hypothesized that the CeA-Vme pathway regulates stress-induced masseter muscle overactivity. An animal model was established by exposing mice to chronic restraint stress (CRS). We tested the electromyography activity of the masseter muscle, as the masseter is the main muscle responsible for elevating the jaw during oromotor movements (Kitagawa et al., 2021; Arizono-Shimada et al., 2022). In addition, we used several morphological approaches to confirm the existence of CeA-Vme projections and the neurotransmitters expressed by the projecting neurons and performed functional investigations to explore the involvement of the CeA-Vme pathway in stress-induced overactivity of the masticatory muscles.

## 2 Materials and methods

### 2.1 Animals

C57BL/6J (Laboratory Animal Center of Fourth Military Medical University), glutamic acid decarboxylase (GAD) 1-GFP (Jackson Laboratory, JAX stock #007677), and GAD2-Cre (Jackson Laboratory, JAX stock #010802) mice were used in this study. The C57BL/6 J mice were used for initial behavioral and EMG tests, as well as the retrograde tracing with FG and the anterograde tracing with BDA. The GAD1-GFP mice were used to visualize the GABAergic neurons in neural tracing, immunofluorescence staining, and whole-cell patch-clamp recording experiments. The GAD2-Cre mice were used for neural tracing, chemogenetic and optogenetic experiments. All mice (male, 8–10 weeks of age, 20–25 g) were housed under standard laboratory conditions (12-h light/12-h dark cycle, temperature of 25 ± 1°C, humidity of 50 ± 5%, and *ad libitum* food and water intake.). The Ethics Committee of the School of Stomatology, Fourth Military Medical University, approved all the animal experiments (K9-2022-020).

### 2.2 Restraint stress

An animal model was created by exposing mice to chronic restraint stress (CRS) as described in previous research (Zhao et al., 2022; Herselman et al., 2023). The mice in the CRS group were kept in centrifuge tubes (with ventilation holes) 4 h per day for 14 consecutive days (started at 9:00–11:00 every day). Throughout the CRS protocol, the mice were deprived of food and water. The mice in the control group were raised under normal conditions.

### 2.3 Behavioral tests

The behavioral tests were performed on the next day of the last restraint stress. The anxiety levels of the mice were evaluated via the open field (OF) test and elevated plus maze (EPM) test following methods previously described (Zhao et al., 2022; Segklia et al., 2019). The OF test was conducted by placing the mouse in a cover-free Plexiglas box (50 cm × 50 cm × 45 cm, RD 1412-OF, Shanghai Mobile Datum Co., Ltd., Shanghai, China) under dim illumination. The mouse was allowed to freely explore for 15 min and their activity was monitored by an automated analysis system. All animals were habituated to the testing room for 30 min before the test. The time spent and the distance moved in the center area of the OF were calculated for assessing the anxiety level. A shorter time spent and shorter distance traveled in the central area of the OF indicated higher anxiety levels.

The EPM system (RD1208-EP, Shanghai Mobile Datum Co., Ltd., Shanghai, China) was located 50 cm from the ground and consisted of two opposite open arms (OAs, 30 × 5 cm), two opposite closed arms (CAs, 30 × 5 × 25 cm), and a central activity area (5 × 5 cm). The mice were placed in the central area and allowed to move freely for 5 min. All animals were habituated to the testing room for 30 min before the test and their activities were recorded through a camera and processed with an automatic analysis system. The time spent and the number of entries in the open arms of the mice were calculated for assessing the anxiety level. A shorter time spent and fewer entries in the open arms of the EPM indicated higher anxiety levels.

### 2.4 Electromyographic recordings

To assess muscular activity, we performed EMG recordings of the masseter muscles of mice as described previously with minor modifications (Han et al., 2017; Yu et al., 2021). Firstly, the customized electrodes (KedouBC Technology Co., Ltd, Suzhou, China) were implanted into left muscles of mice before the beginning of the experiment. An insulated flexible wire (diameter: 0.5 mm) was used, with one end was soldered to a stainless-steel microneedle and the other end was soldered to a female connector. After anesthesia with intraperitoneal injection of sodium pentobarbital (35 mg/kg), the skin on the left cheek and the top of the skull was prepared, sterilized and then incised. The skin of the left cheek was incised to expose the masseter muscle, and the electrode microneedle was inserted into the muscle belly and fixed with a suture. The insulated wires walked subcutaneously through the posterior ear and threaded out at the cranial incision, and the female connector was fixed to the cranial vault bone with dental cements. Antibiotic (cefotiam hydrochloride, 66 mg/kg) and analgesic (flurbiprofen axetil, 3.3 mg/kg) were administered intraperitoneally before allowing the mice to recover.

Experiments were started at least 1 week after electrodes implants surgery. On the next day of the last restraint stress, the EMG level of masseter muscles of mice in the awake state were recorded by MP46 physiological recorder (BIOPAC, United States). Before testing, mice were adapted to the environment and connected to the recording connector for 3 days in the sound-proof chamber. The EMG signals of the masseter muscles of the mice in the waking state (30 min for each mouse) was collected. The data were processed with AcqKnowledge software (USA) to obtain the integral electromyography (iEMG) and Root Mean Square (RMS) of the masseter muscles of each mouse.

### 2.5 Stereotaxic injection

The stereotaxic injection was performed as described previously (Yin et al., 2020). Briefly, mice were anesthetized with sodium pentobarbital (35 mg/kg, i.p.), and were fixed on a stereotaxic apparatus (RWD, China). A midline incision was made on the head to fully expose the skull. A hole was drilled through the skull to insert a glass micropipette connected to a microsyringe (1 mL, Hamilton) into the target brain site according to the mouse brain Stereotaxic atlas (Paxinos and Franklin, 2001). The tracer or virus injections were made by pressure with a microinjection pump (RWD, China) at a rate of 20 nL min^−1^ and the pipette was held in place for 15 min after the injection to insure the virus absorption into the tissue and reduction of possible spread.

For retrograde tracing of Vme-Vmo projections, the tracer FG was injected into the Vmo of C57BL/6 J mice. To perform anterograde tracing of CeA-Vme projections, C57BL/6 J mice were injected with BDA into the CeA. For cell type-specific anterograde tracing, first, Cre-dependent rAAV2/9-EF1α-DIO-mCherry-WPRE-hGH was injected into the CeA of GAD2-Cre mice, which allowed mCherry to be expressed in GAD2-containing neurons. Second, rAAV2/1-hSyn-CRE-WPRE and rAAV2/9-EF1α-DIO-mCherry-WPRE were mixed and injected into the CeA of GAD1-GPF mice. Moreover, rAAV2/9-EF1α-DIO-mCherry-WPRE was injected into the ipsilateral Vme of the same GAD1-GPF mice. After recovery and virus expression for 4 weeks, the animals were sacrificed.

For the chemogenetical experiment, Cre-dependent rAAV2/9-EF1α-DIO-hM4D(Gi)-mCherry-WPREs was injected into the CeA of GAD2-Cre mice. For optogenetic manipulation of CeA-Vme GABAergic projections, the retrograde virus rAAV2/R-EF1α-DIO-eNpHR-EYFP-WPRE was injected into the Vme, and an optic fiber was implanted in the CeA. All mice were allowed to recover for 4 weeks before the behavioral tests or EMG recording. Detailed information about the tracer, virus and the injection location are provided in Supplementary Table S1.

### 2.6 Immunofluorescence staining

Immunofluorescence staining was conducted as described previously (Yin et al., 2020). For PV/NeuN or PV staining in Vme, the sections were incubated with anti-NeuN (Abcam) and/or anti-PV (Abcam) primary antibodies. The mice were deeply anesthetized with sodium pentobarbital (60 mg/kg, i.p.) and perfused with 0.1 M phosphate buffer (PB, pH 7.4) containing 4% paraformaldehyde. The whole brain was removed for post-fixation and immersed in 30% sucrose in 0.1 M PB for dehydration. Transverse sections containing CeA or Vme were cut at 35 μm thickness on a cryostat (Leica CM1800, Heidelberg, Germany) at −20°C, and the sections were collected in 0.01 M PBS (pH 7.4). The sections were blocked with 10% donkey serum for 30 min at room temperature, then incubated overnight with primary antibodies. Next, the sections were washed and incubated with the appropriate fluorophore-conjugated secondary antibodies or avidin for 4 h at room temperature. Finally, all sections were mounted on glass slides and observed under a laser confocal microscope (FV1000; Olympus, Tokyo, Japan). The numbers of Fos + and GFP+ neurons in the CeA of GAD1-GFP mice in the control and CRS groups were counted by an investigator who was blinded to the treatment conditions (Effinger et al., 2023). The detailed information about all the antibodies used is provided in Supplementary Table S2.

### 2.7 Whole-cell patch-clamp recording

Whole-cell patch-clamp recordings were performed on GFP+ interneurons or pseudounipolar GFP− neurons in the Vme according to previous study (Zhang et al., 2022). After anesthesia using 2% isoflurane, the GAD1-GFP mice from CON and CRS group were sacrificed and the brains were immediately removed and placed in oxygenated (95% O_2_ and 5% CO_2_) ice-cold artificial cerebrospinal fluid (ACSF) containing 124 mM NaCl, 2.5 mM KCl, 2 mM MgSO_4_ + 7H_2_O, 2 mM CaCl_2_, 1 mM NaH2PO_4_, 25 mM NaHCO_3_, 25 mM glucose, 1 mM ascorbate, and 3.0 mM pyruvate. Transverse slices (300 μm) containing the Vme were cut on a Leica vibratome (Leica VT 1200s, Heidelberger, Nussloch, Germany) at 4°C and transferred to a chamber with oxygenated ACSF and recovered for 1 h in room temperature. The whole-cell patch-clamp recordings were performed in current-clamp or voltage-clamp mode using an Axon 700B amplifier (Molecular Devices, Forster City, CA, United States) on GFP+ interneurons or GFP− pseudounipolar neurons in Vme that were visualized under epifluorescence using a filter set (U-HGLGPS, Olympus) with a monochrome CCD camera (IR-1000E, DAGE-MTI) and monitor. 0.2% biocytin (MilliporeSigma) was introduced into the recording solution to identify the recorded neurons. In current clamp mode, the action potentials (APs) of neurons were evoked by intracellular injection of 0–200-pA depolarizing currents in increments of 25 pA for 400 ms. In voltage clamp mode, inhibitory postsynaptic currents (IPSC) were recorded 3 min at a holding potential of 0 mV and the recording pipettes were filled with solution containing 112 mM Cs-gluconate, 5 mM TEA-Cl, 3.7 mM NaCl, 0.2 mM EGTA, 10 mM HEPES, 2 mM MgATP, 0.3 mM Na3GTP and 5 mM QX-314 (adjusted to pH 7.2 with CsOH, 290 mOsmol). Clampex and Clampfit 10.2 software (Molecular Devices, Forster City, CA, United States) were used to acquire and analyze the data. After the recording finished, 0.2% biocytin (Sigma) was added to the recording solution to identify the recorded neurons. The slices were post-fixed and dehydrated, and subjected to staining of biocytin-filled neurons.

### 2.8 Chemogenetic and optogenetic manipulation

For chemogenetic manipulation, the mice were first subjected to virus injection and/or EMG electrode implantation. After 2 weeks, the stress protocol began. After the last exposure to restraint stress, the mice received an i.p. injection of 1 mg/kg clozapine N-oxide (CNO, Sigma) or 50 μL saline, followed by EMG recording of the masseter muscle, the OF test and EPM test.

For optogenetic manipulation, the mice were subjected to virus injection, optic fiber implantation, and/or EMG electrode implantation. After 2 weeks, the stress protocol began. After the last exposure to restraint stress, mice were stimulated with light by connecting the ferrule to a fiber optic cable via a rotary joint (Inper) and then connecting it to another fiber optic cable that was linked to a fiber-coupled steady 589 nm yellow laser (8 mW/mm^2^, Inper). The OF test and EMG recordings lasted 15 min in total, with a 5-min period before stimulation, a 5-min period during stimulation, and a 5-min period after stimulation. During the EPM test, the behavior of the mice was observed for a duration of 9 min, i.e., three 3-min segments of light offs, lights on, and lights off (Chiou et al., 2016). At the end of the experiments, all the mice were perfused, and tissues were collected and sectioned for visualization of the CeA and Vme. Animals in which the injection site or optic fiber implantation site was incorrect were excluded.

### 2.9 Statistical analysis

All experimental data are expressed as the means ± SDs. Statistical analysis was performed via SPSS 22.0 software (SPSS Inc., Chicago, IL, United States). Unpaired Student’s *t*-test, one-way ANOVA, and two-way repeated-measures ANOVA were used to assess statistical significance. Bonferroni’s *post-hoc* test was conducted after ANOVA. Statistical significance was defined as *p* < 0.05. All the data are available from the corresponding authors on reasonable request.

## 3 Results

### 3.1 Anxiety-like behavior and masseter overactivity were induced by stress

**Figure 1:**
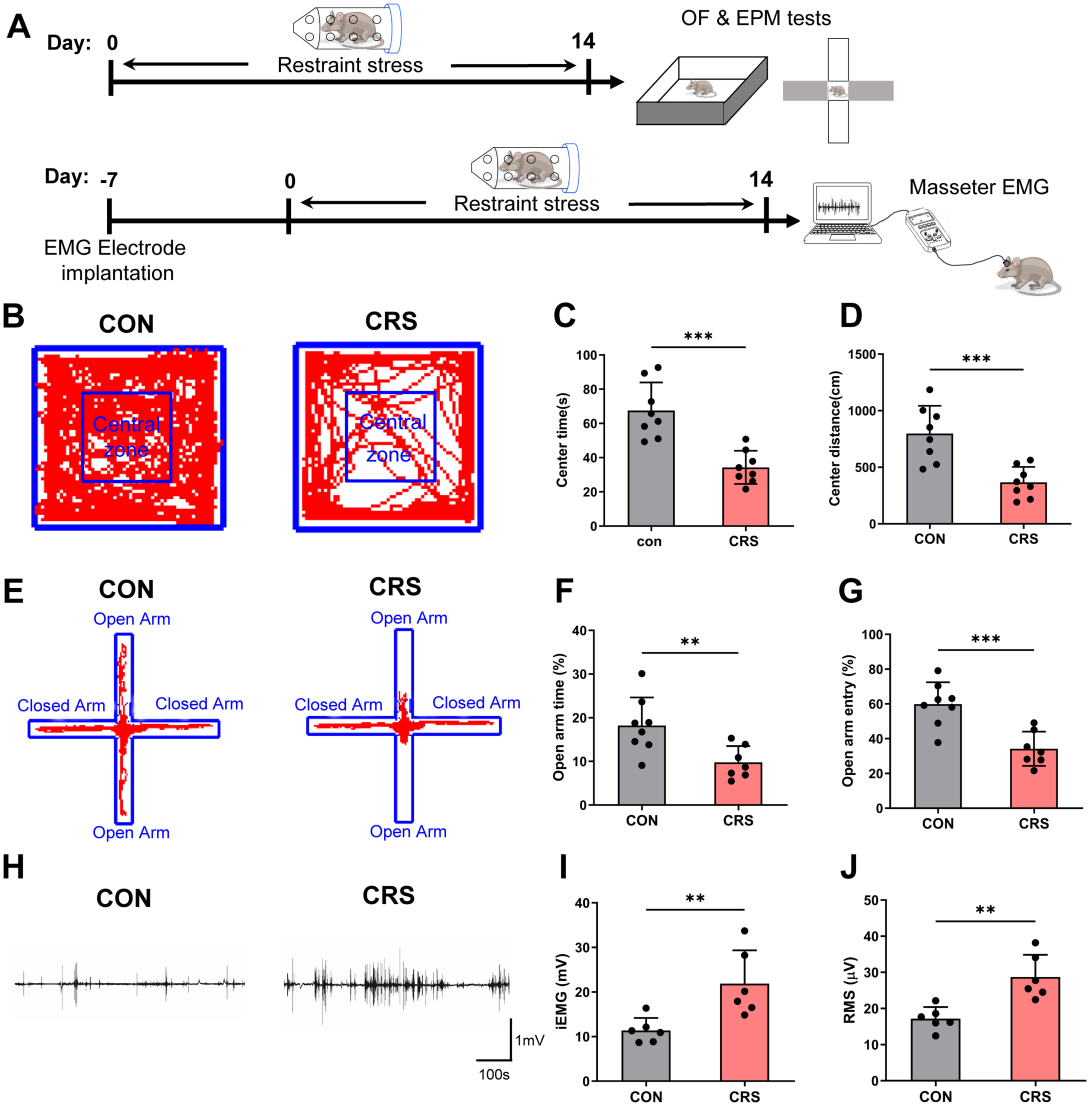
Anxiety-like behavior and masseter muscle overactivity are induced by stress. (A) Schematic of the experimental procedure. (B) Representative trajectories of mice in the control and CRS groups in the OF test (CON, *n* = 8; CRS, *n* = 8). CON: control group. The time spent (C) and distance traveled (D) in the central area of the OF were significantly lower in the CRS group than in the control group (unpaired Student’s *t*-test). (E) Representative trajectories of mice in the control and CRS groups in the EPM test (CON, *n* = 8; CRS, *n* = 7). The percentage of time spent in (F) and percentage of entries (G) into the open arm were significantly lower in the CRS group than in the control group (unpaired Student’s *t*-test). (H) Representative EMG waveforms of the masseter muscles of mice in the control and CRS groups (CON, *n* = 6; CRS, *n* = 6). The iEMG signals (I) and RMS values (J) of mice in the CRS group were significantly greater than those of mice in the control group (unpaired Student’s *t*-test). **p < 0.01, ***p < 0.001.

To assess the impacts of stress on the emotional status and masseter activity, mice were exposed to CRS for 14 days, followed by behavioral tests and electromyographic (EMG) recordings of masseter (Figure 1A). The anxiety levels of the mice were evaluated via the open field (OF) test and elevated plus maze (EPM) test. The results revealed that the mice in the CRS group spent less time and traveled shorter distances in the center area of the OF (Figures 1B–D) and spent less time and made fewer entries in the open arm of the EPM than those in the control group did (Figures 1E–G), indicating that CRS elevated anxiety levels in the mice.

Furthermore, as to the masseter EMG results, compared with those in the control group, mice the CRS group presented significantly increased integral electromyography (iEMG) amounts and higher Root Mean Square (RMS) values of the masseter muscles (Figures 1H–J). These results indicate that CRS can lead to increased masseter muscle activity in mice.

### 3.2 The CeA sends GABAergic projections to the Vme

**Figure 2:**
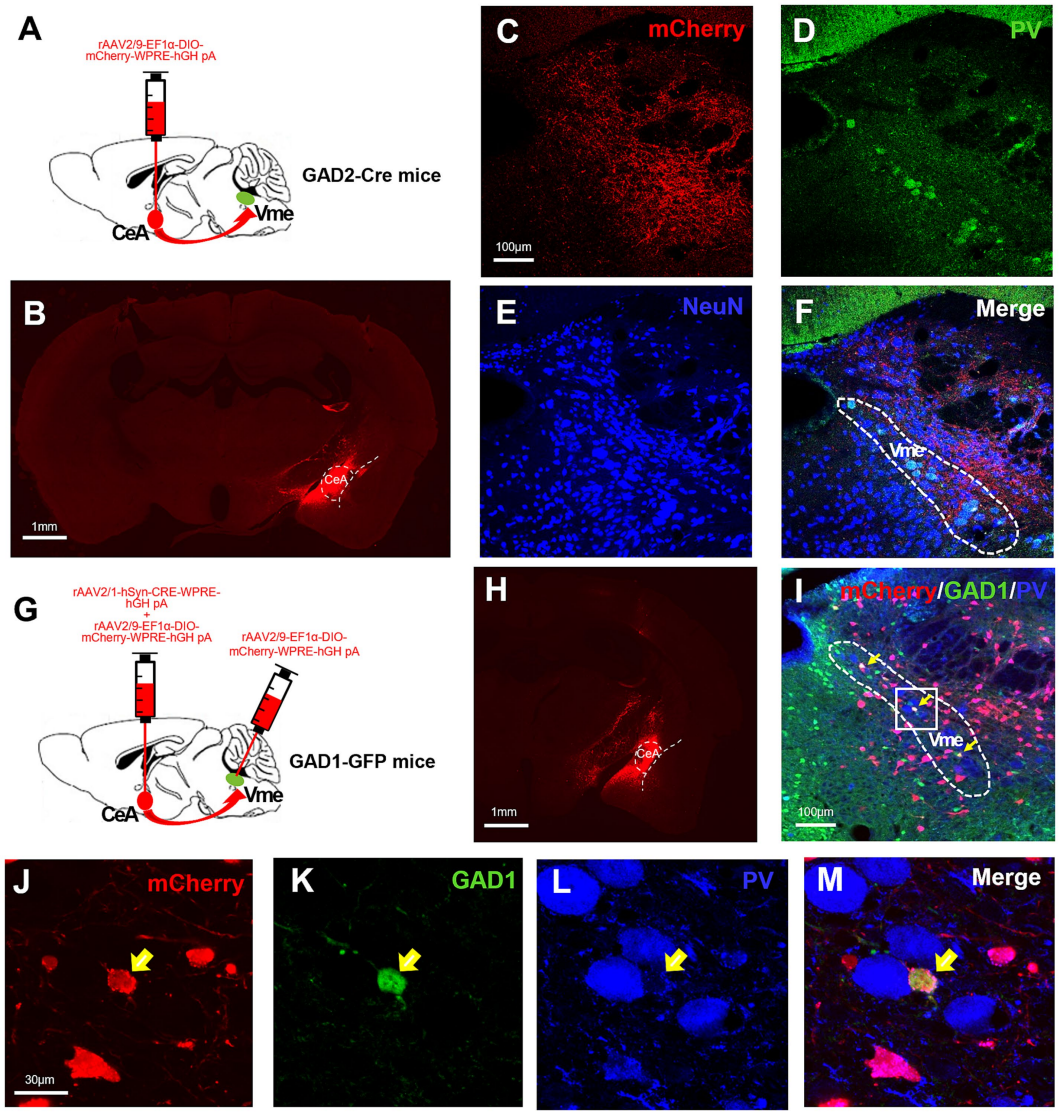
The CeA sends GABAergic projections to the Vme, and these projections terminate on GABAergic neurons within the Vme. (A) Schematic of the injection of the anterograde virus rAAV2/9-EF1α-DIO-mCherry-WPRE into the CeA of GAD2-Cre mice (*n* = 3). (B) The injection site in the CeA. Scale bar = 1 mm. (C–F) mCherry-labeled neural terminals were observed in the Vme (where pseudounipolar PV+ neurons were located), indicating direct CeA-Vme GABAergic neural projections. Scale bar = 100 μm. (G) Schematic showing the injection of the transsynaptic anterograde virus rAAV2/1-hSyn-CRE-WPRE mixed with rAAV2/9-EF1α-DIO-mCherry-WPRE into the CeA and rAAV2/9-EF1α-DIO-mCherry-WPRE into the ipsilateral Vme of the same GAD1-GPF mice (*n* = 3). (H) The injection site in the CeA. Scale bar = 1 mm. (I) The mCherry-labeled neurons in the Vme were GFP+ but not PV+ (yellow arrows in I–M), suggesting that the projections from the CeA terminated on GABAergic interneurons within the Vme but not pseudounipolar neurons. Scale bar = 100 μm. (J–M) Magnified image of the framed area in (I). Scale bar = 30 μm.

Given the important roles of the CeA in stress-related behaviors and the Vme in the control of jaw muscle movement, we explored the direct projections from CeA to Vme. For retrograde tracing of Vme-Vmo projections, the tracer Fluoro-gold (FG) was injected into the Vmo of C57BL/6 J mice. The FG-labeled neurons in Vme were large pseudounipolar neurons that were also PV+ (Supplementary Figure S1). To perform anterograde tracing of CeA-Vme projections, the anterograde tracer biotinylated dextran amine (BDA) was injected into the CeA, and dense BDA-labeled fibers and terminals were observed in the Vme (Supplementary Figure S2). The results suggested that there are direct projections from CeA to Vme and from Vme to Vmo. To further verify the nature of the CeA-Vme projections, Cre-dependent anterograde labeling virus rAAV2/9-EF1α-DIO-mCherry-WPRE-hGH was injected into the CeA of GAD2-Cre mice (Figures 2A,B). The mCherry labeled neural terminals were seen in Vme site, where the PV+ pseudounipolar neurons distribute (Figures 2C–F). These results indicated that there exists a direct CeA-Vme GABAergic neural projection.

### 3.3 The CeA projects to GABAergic interneurons in the Vme

To confirm the cell type in the Vme that receives projections from the CeA, we injected the anterograde transsynaptic labeling virus rAAV2/1-hSyn-CRE-WPRE-hGH into the CeA and simultaneously injected rAAV2/9-EF1α-DIO-mCherry-WPRE-hGH into the ipsilateral Vme of GAD1-GFP mice (Figure 2G). The injection sites of CeA were testified by injecting rAAV2/9-EF1α-DIO-mCherry-WPRE-hGH into CeA at the same time (Figure 2H). After 4 weeks, the mCherry labeled neurons were observed in Vme and those neurons were also GFP+ rather than PV+ (Figures 2I–M). These results suggest that the projections from the CeA terminate on GABAergic interneurons within the Vme but not pseudounipolar PV+ neurons.

### 3.4 CRS induces the activation of CeA neurons and opposite changes in the excitability of multipolar interneurons and pseudounipolar neurons in the Vme

**Figure 3:**
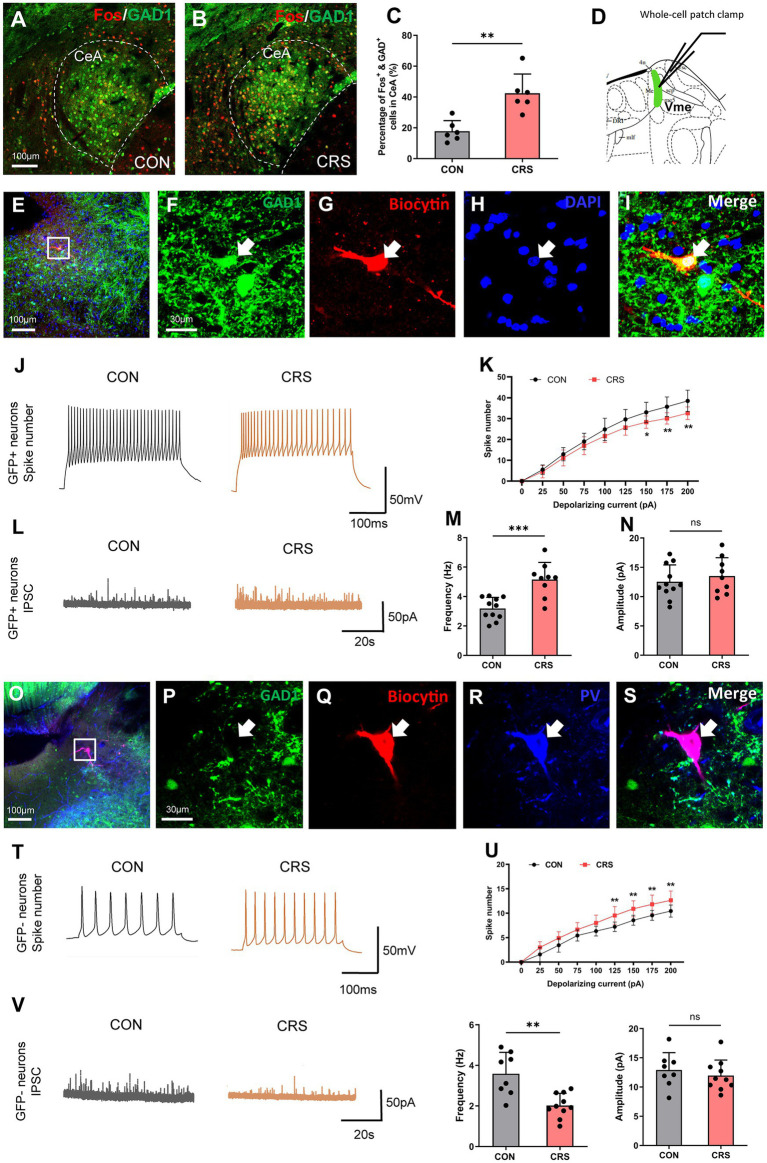
CRS induces the activation of CeA neurons and opposite changes in the excitability of multipolar interneurons and pseudounipolar neurons in the Vme. (A,B) Immunofluorescence staining of Fos in the CeA of GAD1-GFP mice. Scale bar = 100 μm. (C) The numbers of Fos + and GFP+ neurons in the CeA were significantly greater in the CRS group than in the control group (CON, *n* = 6; CRS, *n* = 6. unpaired Student’s *t*-test). (D) Whole-cell patch-clamp recordings were used to assess the excitability of two types of neurons in the Vme of GAD1-GFP mice. (E–I) The biocytin-filled neurons were also GFP+, indicating that the recorded neurons were GABAergic interneurons. Scale bar = 100 μm (E); Scale bar = 30 μm (F–I). (J) Representative recordings of GABAergic interneurons in the Vme of mice in the control and CRS groups after the delivery of 150 pA currents to cells (CON, *n* = 10; CRS, *n* = 10). (K) The firing rates of GABAergic interneurons in the Vme were significantly lower in the CRS group than in the control group when currents of 150, 175, and 200 pA were delivered (two-way repeated-measures ANOVA with Bonferroni’s *post-hoc* test). (L) Representative diagrams of IPSCs of GABAergic interneurons in the Vme of mice in the control and CRS groups (control, *n* = 11; CRS, *n* = 9). (M,N) The IPSC frequency was significantly greater in the CRS group than in the control group, whereas the amplitude was not significantly different (unpaired Student’s *t*-test). (O–S) The biocytin-filled neurons were also PV+, indicating that the recorded neurons were pseudounipolar neurons. Scale bar = 100 μm (O); Scale bar = 30 μm (P–S). (T) Representative recordings of pseudounipolar neurons in the Vme in the control and CRS groups after the delivery of 150 pA currents into the cells (CON, *n* = 9; CRS, *n* = 11). (U) The firing rates of pseudounipolar neurons in the Vme were significantly greater in the CRS group than in the control group when currents of 125, 150, 175, and 200 pA were delivered (two-way repeated-measures ANOVA with Bonferroni’s *post-hoc* test). (V) Representative diagrams of IPSCs of pseudounipolar neurons in the Vme in the control and CRS groups (CON, *n* = 8; CRS, *n* = 10). (W,X) The frequency was significantly lower in the CRS group than in the control group, while the amplitude was not significantly different (unpaired Student’s *t*-test). **p < 0.01, ***p < 0.001, NS, not significant (*p* > 0.05).

To investigate the impact of CRS on the activity and excitability of CeA and Vme neurons, we conducted immunofluorescence staining of Fos in CeA and whole-cell patch-clamp recordings in the Vme of GAD1-GFP mice. The findings revealed a notable increase in the quantity of both Fos + and GFP+ neurons in the CeA in mice in the CRS group compared with those in the control group, suggesting that CRS could trigger the activation of GABAergic neurons in the CeA (Figures 3A–C).

Whole-cell patch-clamp recordings were used to measure the responsiveness of two different neuron types including big pseudounipolar neurons and small multipolar GABAergic interneurons in the Vme (Figure 3D, 5.34 mm caudal to Bregma, Paxinos and Franklin, 2001). The findings indicated that GABAergic interneurons presented fewer spikes in the CRS group compared to the control group in response to currents of 150, 175, and 200 pA (Figures 3E–K). As the CeA sends GABAergic neural projections to GABAergic interneurons in the Vme, we further recorded the IPSCs of GABAergic interneurons in the Vme and found that the IPSC frequency was higher in the CRS group than in the control group (Figures 3L,N). Conversely, there was a notable increase in the number of spikes produced by large pseudounipolar neurons in the Vme in the CRS group compared with the control group in response to currents of 125, 150, 175, and 200 pA (Figures 3O–U). Moreover, the IPSC frequency of pseudounipolar neurons in the Vme was significantly lower in the CRS group than in the control group (Figures 3V–X). These results suggest that CRS could lead to inhibited excitability of small multipolar GABAergic interneurons but increased excitability of large pseudounipolar neurons in the Vme.

### 3.5 Reducing the activity of GABAergic neurons in the CeA relieves anxiety and muscle overactivity caused by restraint stress

**Figure 4:**
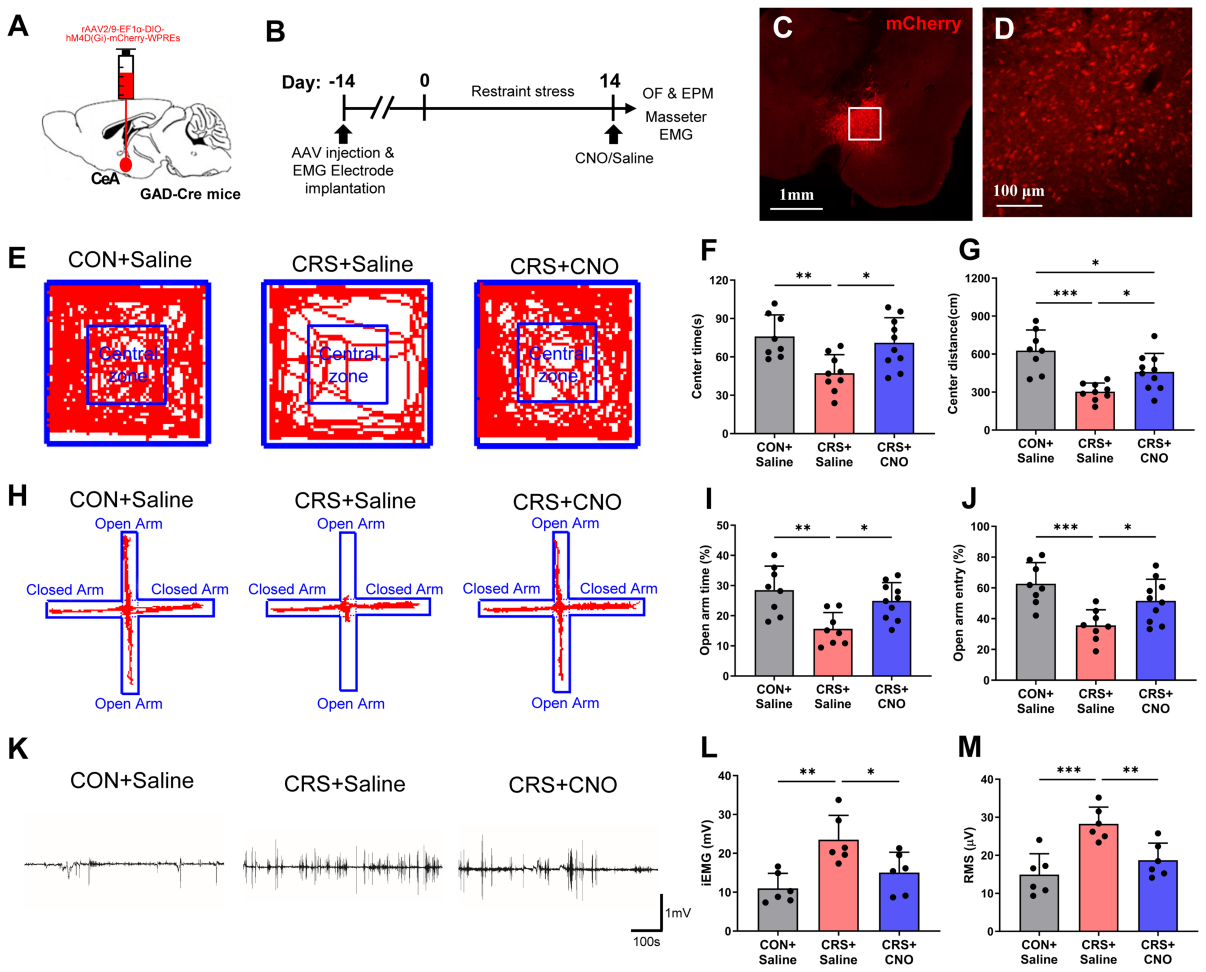
Reducing the activity of GABAergic neurons in the CeA relieves anxiety and muscle overactivity caused by restraint stress. (A) Schematic showing the injection of rAAV2/9-EF1α-DIO-hM4D(Gi)-mCherry-WPREs into the CeA of GAD2-Cre mice. (B) Schematic diagrams of the experimental procedures for chemogenetics manipulation. (C) The injection site of the CeA. Scale bar = 1 mm. (D) Magnified image of the framed area in (C). Scale bar = 100 μm. (E) Representative trajectories of mice from each group in the OF test (CON + saline, *n* = 8; CRS + saline, *n* = 9; CRS + CNO, *n* = 10). (F,G) The time spent and distance traveled in the central area of the OF were significantly shorter in the CRS + saline group than in the control + saline group, whereas these parameters were significantly greater in the CRS + CNO group than in the CRS + saline group (one-way ANOVA with Bonferroni’s *post-hoc* test). (H) Representative trajectories of mice from each group in the EPM test (CON + saline, *n* = 8; CRS + saline, *n* = 8; CRS + CNO, *n* = 10). (I,J) The time spent in and number of entries into the open arm were significantly lower in the CRS + saline group than in the control + saline group, whereas these parameters were significantly greater in the CRS + CNO group than in the CRS + saline group (one-way ANOVA with Bonferroni’s *post-hoc* test). (K) Representative EMG waveforms of the masseter muscles of the mice in each group (CON + saline, *n* = 6; CRS + saline, *n* = 6; CRS + CNO, *n* = 6). (L,M) The iEMG signal and RMS value of the masseter muscle were significantly greater in CRS + saline group than in the control + saline group, whereas these parameters were significantly lower in the CRS + CNO group than in the CRS + saline group (one-way ANOVA with Bonferroni’s *post-hoc* test). *p < 0.05, **p < 0.01, ***p < 0.001.

Given that GABAergic neuron activation in the CeA is triggered by CRS, we used chemogenetic approaches to investigate the roles of CeA GABAergic neurons in CRS-evoked anxious behaviors and masseter overactivity (Figures 4A,B). The injection site and validity of the virus transfection was verified by mCherry expression in CeA neurons (Figures 4C,D). The findings indicated that mice in the CRS + Saline group exhibited significantly decreased time spent and shorter moving distance in center area in the OF test compared with the mice in the CON+saline group, whereas the CRS + CNO group had an increased time spent and distance moved in center area compared to the mice in the CRS + saline group (Figures 4E–G). Furthermore, decreased percentages of OA retention time and OA entries were observed in CRS + Saline group compared with the CON+Saline group in EPM test whereas CRS + CNO group exhibited an increased percentages of OA retention time and OA entries compared to the mice in the CRS + saline group (Figures 4H–J). These results revealed that specifically reducing GABAergic neuron activity within the CeA could alleviate anxiety-like behaviors initiated by stress.

Similarly, the results of masseter EMG recordings revealed that the iEMG and RMS levels of the masseter muscle of the mice in CRS + Saline group were significantly higher than those of the CON+saline group, whereas the iEMG and RMS levels of the masseter muscle of the mice in CRS + CNO group were significantly lower than those of the CRS + saline group (Figures 4K–M). These findings indicate that blocking the stimulation of GABAergic neurons in the CeA could reduce the overactivity of the masseter muscle caused by CRS.

### 3.6 Specific inhibition of the CeA-Vme GABAergic pathway does not alleviate CRS-induced anxiety-like behavior

**Figure 5:**
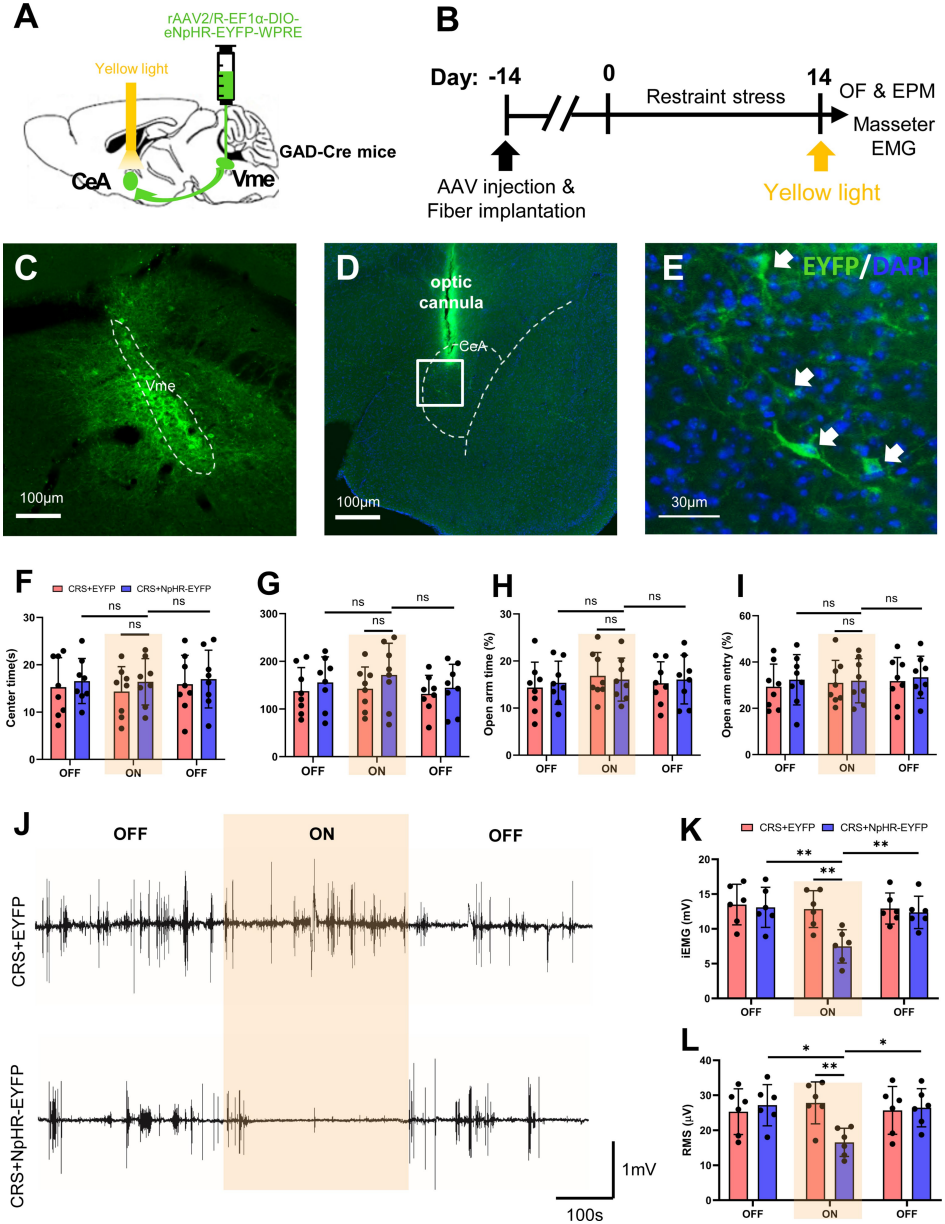
Specific inhibition of the CeA-Vme GABAergic pathway reverses masseter muscle overactivity initiated by stress. (A) Schematic showing the injection of the retrograde virus rAAV2/R-EF1α-DIO-eNpHR-EYFP-WPRE into the Vme of GAD2-Cre mice and the implantation of optic fibers in the ipsilateral CeA. (B) Schematic diagrams of the experimental procedures for optogenetic manipulation. (C) The injection site in the Vme. Scale bar = 100 μm. (D,E) The EYFP-labeled cell bodies of neurons were observed in the ipsilateral CeA. The framed area in (D) is magnified in (E). The white arrows indicate labeled neurons. Scale bar = 100 μm (D). Scale bar = 30 μm (E). (F–I) Specific inhibition of the CeA-Vme GABAergic pathway did not affect anxiety levels, as anxiety levels were not different not only between the CRS + HpHR-EYFP group and the CRS + EYFP group, but before and after light stimulation in the CRS + HpHR-EYFP group (OF and EPM: CRS + EYFP, *n* = 8; CRS + HpHR-EYFP, *n* = 8. two-way repeated-measures ANOVA with Bonferroni’s *post-hoc* test). (J) Representative EMG waveforms of masseter muscles in the CRS + EYFP and CRS + HpHR-EYFP groups when light was turned off, then on and then off (CRS + EYFP, *n* = 6; CRS + HpHR-EYFP, *n* = 6). (K,L) Masseter muscle activity was significantly inhibited when the light was on compared to when the light was off in the CRS + HpHR-EYFP group and when the light was on in the CRS + HpHR-EYFP group compared to in the CRS + EYFP group (two-way repeated-measures ANOVA with Bonferroni’s *post-hoc* test). *p < 0.05, **p < 0.01, ***p < 0.001, NS, not significant (*p* > 0.05).

Given the direct projections from the CeA to the Vme and activation or excitability changes of neurons in CeA or Vme caused by stress, optogenetic approaches were utilized to explore the role of CeA to Vme GABAergic projections in the increase in anxiety levels and masseter muscle activity elicited by CRS (Figures 5A–E). The behavioral results revealed that regardless of whether light stimulation was delivered, the mice in both CRS + EYFP group and CRS + HpHR-EYFP group showed obviously less time spent and shorter moving distance in the center area in OF test and lower percentage of retention time in OAs and entries into OAs compared to those in the CON+EYFP group. Furthermore, no significant difference of the aforementioned parameters was found between CRS + EYFP group and CRS + HpHR-EYFP mice after light stimulation (Figures 5F–I). These results reveal that targeting the CeA-Vme GABAergic pathway does not reduce anxiety-like behaviors caused by CRS.

### 3.7 Specific inhibition of the CeA-Vme GABAergic pathway reverses masseter muscle overactivity initiated by stress

EMG recording during optogenetic manipulation revealed that when the stimulating light was off, the iEMG and RMS levels of masseter muscle in CRS + EYFP group and CRS + HpHR-EYFP group were significantly higher than those in CON+saline group, and no statistical significance of the masseter iEMG and RMS levels was found between CRS + EYFP group and CRS + HpHR-EYFP mice. However, the yellow light stimulation significantly lowered the iEMG and RMS levels of masseter muscle in CRS + HpHR-EYFP group compared with those in the CRS + EYFP group (Figures 5J–L). These findings indicate that blocking the activation of the CeA-Vme GABAergic pathway can reduce the overactivity of the masseter muscle induced by CRS.

## 4 Discussion

Clinical studies have indicated that stress, anxiety, and other negative emotions are closely related to masticatory muscle overactivity and chronic muscle pain (Janal et al., 2021; Silva et al., 2022; Solis et al., 2024), indicating a possible causal link between stress and masticatory muscle hyperactivity, which may be responsible for painful TMD due to continuous overloading of the muscles and temporomandibular joint (Abe et al., 2013; Nickel et al., 2022; Takashima et al., 2017). In this study, the EMG signal of the masseter muscle was significantly elevated in mice with chronic stress-induced anxiety, confirming the existence of a causal relationship between stress and masticatory muscle hyperactivity. Therefore, further exploration of the central mechanisms of this relationship is necessary.

The amygdala complex in the central nervous system is closely related to emotional processing; the lateral/basolateral amygdala receives information from the cortex, thalamus and other brain areas and is transmits the information into the CeA, which projects information through efferents, producing physiological and behavioral responses to stress (Kim et al., 2017; Gunduz-Cinar et al., 2023). In the present study, the results reconfirmed the significant activation of GABAergic neurons in the CeA in mice with anxiety symptoms, suggesting the potential role of the CeA in the process of anxiety regulation (Babaev et al., 2018; Whittle et al., 2021; Pomrenze et al., 2019).

The Vme plays an important role in the central control of oral movements, and it is the key nucleus that connects proprioceptive afferents from orofacial muscles and periodontal membrane and regulates the oromotor reflex through central feedback processing (Yoshida et al., 2017; Fortin et al., 2021). Given that stress can induce masseter overactivity, we further studied Vme neurons and found that the excitability of neurons in the Vme was significantly elevated under stress conditions and the masseter overactivity was notably alleviated after inhibition of glutamatergic neuron excitability in the Vme (Zhao et al., 2022). Owing to the central location of the Vme, which allows it to receive neural projections from higher nuclei in the forebrain to modulate its neuronal activity (Lazarov et al., 2011; Shirasu et al., 2011; Mascaro et al., 2009; Ohara et al., 2016), the Vme may serve as a pathway through which stress can influence masseter muscle activity.

Although previous studies have demonstrated the existence of direct projections from the CeA to the Vme (Lazarov et al., 2011; Shirasu et al., 2011), the properties of these projections and whether they are directly related to stress-induced jaw muscle overactivity remain unclear. In the present study, we first confirmed the existence of CeA-Vme projections via neural tracing; second, we performed anterograde neural tracing in GAD2-Cre mice and demonstrated that the neurons projecting from the CeA to the Vme are GABAergic (inhibitory). Furthermore, we used a transsynaptic viral labeling technique and reported that the projections from the CeA terminate on GABAergic interneurons in the Vme but not pseudounipolar PV+ neurons. We subsequently used electrophysiological techniques to examine the excitability and IPSCs of the two types of neurons in the Vme after chronic stress. The results revealed that pseudounipolar neurons presented a significant increase in excitability and a decrease in IPSC frequency, whereas GABAergic interneurons presented a decrease in excitability and an increase in IPSC frequency. Overall, we speculate that the increased excitability of excitatory pseudounipolar PV+ neurons in Vme may be a result of disinhibition effect due to the inhibition of GABAergic interneurons in Vme caused by CeA projecting GABAergic neurons.

Furthermore, we employed chemogenetics and optogenetics approaches to detect the role of GABAergic neurons in CeA and GABAergic projections from the CeA to Vme in the process of stress-induced anxiety-like behavior and masseter muscle overactivity. The results showed that inhibiting the activation of GABAergic neurons within the CeA alleviated both stress-induced anxiety and masseter muscle overactivity. In contrast, specific inhibition of GABAergic projections from the CeA to the Vme alleviated stress-induced masseter muscle overactivity but not anxiety-like behavior. We propose that this may be because the central mechanisms associated with anxiety-like behavior are more complex and involve more forebrain regions, including the cortex and thalamus (Kirouac, 2021; Kenwood et al., 2022). Moreover, the failure of anxiety-like behavior relief by optogenetic inhibition of CeA-Vme GABAergic projections may be attributed to a small number of neurons being targeted by the retrograde viruses injected into the Vme. In the future studies, the proportion and the properties of GABAergic neurons in the CeA that project to the Vme may need to be further clarified to explain the results observed in the present study.

It is a limitation in this study that the sample size for the excitability detection of the two different types of neurons in Vme is relatively small. Future studies with larger sample sizes are needed to further validate the role of GABAergic inhibitory interneurons in Vme as well as GABAergic neurons in CeA projecting onto Vme in the process of masseter muscle hyperactivity induced by chronic stress.

In conclusion, the present study revealed that the enhancement of GABAergic neural projections from the CeA to the Vme is one of the important mechanisms by which the central nervous system translates stress information into jaw movements. This study suggests a novel central neural mechanism linking psychological factors with TMDs.

## Data Availability

The datasets presented in this study can be found in online repositories. The names of the repository/repositories and accession number(s) can be found in the article/Supplementary material.
