# Supplementary material for: Regulation of CeA-Vme projection in masseter hyperactivity caused by restraint stress
Source: Front Cell Neurosci. 2024 Nov 21;18:1509020. doi: 10.3389/fncel.2024.1509020 (PMC11617152; doi:10.3389/fncel.2024.1509020)
Supplement: Supplementary file 1 [file Image_1.pdf]

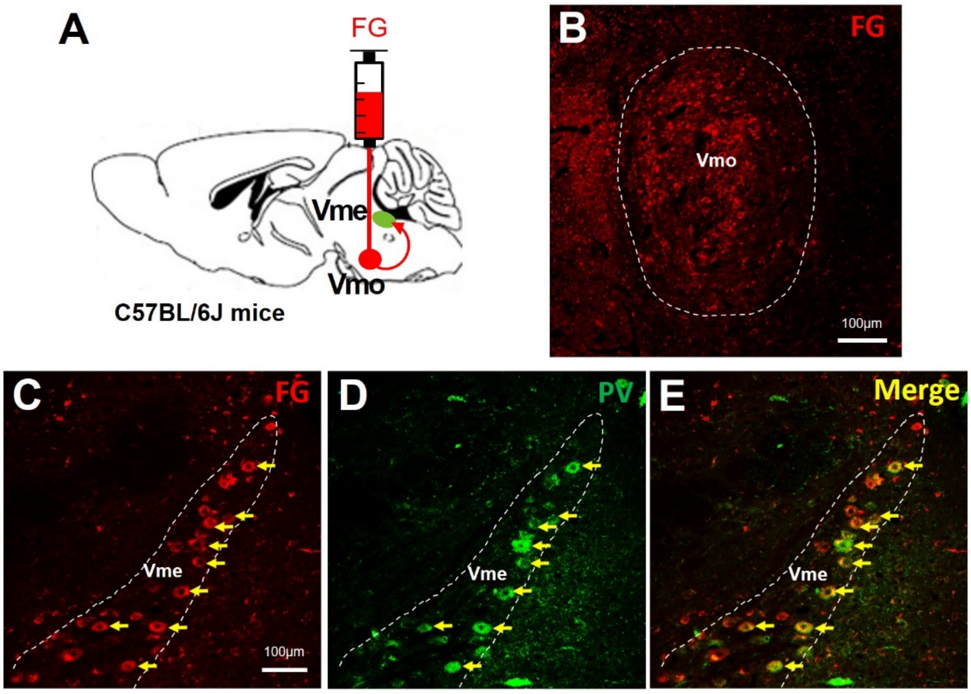

Supplementary Figure 1. Retrograde tracing of Vme-Vmo projections (n=3). (A-B) FG was injected into Vmo. (C-E) The FG^+ neurons in the Vme were large pseudounipolar neurons that were also PV^+ (yellow arrows), suggesting that there are direct Vme-Vmo projections. Scale bars = 100 μm.
