# Supplementary material for: Regulation of CeA-Vme projection in masseter hyperactivity caused by restraint stress
Source: Front Cell Neurosci. 2024 Nov 21;18:1509020. doi: 10.3389/fncel.2024.1509020 (PMC11617152; doi:10.3389/fncel.2024.1509020)
Supplement: Supplementary file 2 [file Image_2.pdf]

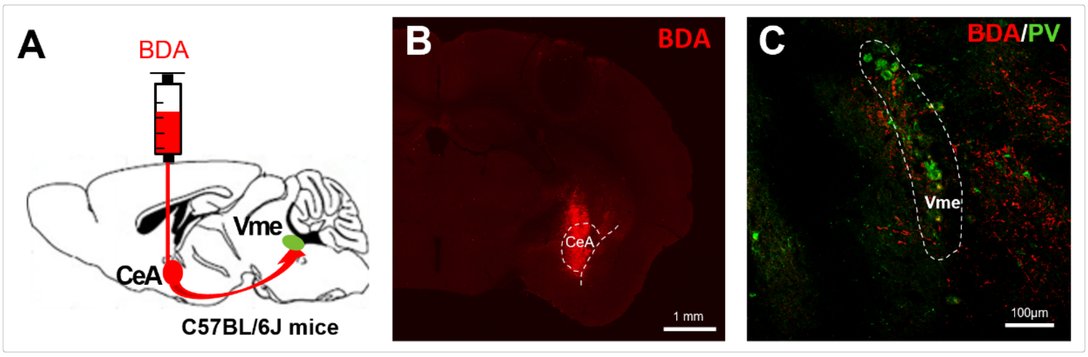

Supplementary Figure 2. Anterograde tracing of CeA-Vme projections (n=3). (A-B) BDA was injected into CeA. Scale bar = 1 mm in (B). (C) the BDA-labeled neural terminals were observed in the Vme (where pseudounipolar PV^+ neurons were located), indicating direct CeA-Vme neural projections. Scale bars = 100 μm.
