# Supplementary material for: Regulation of CeA-Vme projection in masseter hyperactivity caused by restraint stress
Source: Front Cell Neurosci. 2024 Nov 21;18:1509020. doi: 10.3389/fncel.2024.1509020 (PMC11617152; doi:10.3389/fncel.2024.1509020)
Supplement: Supplementary file 3 [file Table_1.DOCX]

**Supplementary Table 1 Detailed information of tracer and virus injection**

| **Item** | **Nuclei (Location)** | **Purpose** | **Mice** | **Volume** | **Manufacture** |
| --- | --- | --- | --- | --- | --- |
| 4% FG | Vmo  (AP:−5.02 mm; ML: +1.40 mm; DV: −4.40 mm) | Retrograde tracing of Vme-Vmo projections | C57BL/6J mice | 0.04 μl | Biotium;  Hayward, CA,  USA |
| 10% BDA | CeA (AP: −1.22 mm; ML: +2.55 mm; DV: −4.70 mm) | Anterograde tracing of CeA-Vme projections | C57BL/6J mice | 0.1 μl | Invitrogen, Eugene, OR, USA |
| rAAV2/9-EF1α-DIO-mCherry-WPRE-hGH | CeA(AP: −1.22 mm; ML: +2.55 mm; DV: −4.70 mm) | Cell type-specific anterograde tracing of CeA-Vme projections | GAD2-Cre mice | 0.2 μl | BrainVTA, Wuhan, China |
| rAAV2/1-hSyn-CRE-WPRE | CeA(AP: −1.22 mm; ML: +2.55 mm; DV: −4.70 mm) | Anterograde transsynaptic tracing of CeA-Vme projections | GAD1-GFP mice | 0.15 μl | BrainVTA, Wuhan, China |
| rAAV2/9-EF1α-DIO-mCherry-WPRE |  |  |  | 0.15 μl | BrainVTA, Wuhan, China |
| rAAV2/9-EF1α-DIO-mCherry-WPRE | Vme  (AP: −5.52 mm; ML: +1.00 mm; DV: −3.50 mm) |  |  | 0.05 μl | BrainVTA, Wuhan, China |
| rAAV2/9-EF1α-DIO-hM4D(Gi)-mCherry-WPREs | CeA(AP: −1.22 mm; ML: +2.55 mm; DV: −4.70 mm) | Chemogenetical experiment | GAD2-Cre mice | 0.2 μl | BrainVTA, Wuhan, China |
| rAAV2/R-EF1α-DIO-eNpHR-EYFP-WPRE | Vme  (AP: −5.52 mm; ML: +1.00 mm; DV: −3.50 mm) | Optogenetic experiment | GAD2-Cre mice | 0.2 μl | BrainVTA, Wuhan, China |
