# Supplementary material for: Regulation of CeA-Vme projection in masseter hyperactivity caused by restraint stress
Source: Front Cell Neurosci. 2024 Nov 21;18:1509020. doi: 10.3389/fncel.2024.1509020 (PMC11617152; doi:10.3389/fncel.2024.1509020)
Supplement: Supplementary file 4 [file Table_2.docx]

**Supplementary Table 2 Detailed information of antibodies**

| Antigen | Primary Antibodies | Secondary Antibodies |
| --- | --- | --- |
| PV/NeuN | mouse anti-PV (1:500; Millipore) / rabbit anti-NeuN (1:500; Abcam) | Alexa488-conjugated donkey anti-mouse IgG (1:500; Thermo Fisher Scientific) / Alexa647-conjugated donkey anti-rabbit IgG (1:500; Thermo Fisher Scientific) |
| PV | mouse anti-PV (1:500; Millipore) | Alexa647-conjugated donkey anti-mouse IgG (1:500; Thermo Fisher Scientific) |
| Fos | mouse anti-Fos (1:500; Abcam) | Alexa594-conjugated donkey anti-mouse IgG (1:500; Thermo Fisher Scientific) |
| Biocytin/DAPI | NA | Alexa594-conjugated avidin (1:1000, Vector Laboratories) / DAPI (1:1000; Sigma) |
| Biocytin/PV | NA / mouse anti-PV (1:500; Millipore) | Alexa594-conjugated avidin (1:1,000, Vector  Laboratories) / Alexa647-conjugated donkey anti-mouse IgG (1:500; Thermo Fisher Scientific) |
| FG/PV | rabbit anti-FG (1:500; Millipore)/ mouse anti-PV (1:500; Millipore) | Alexa594-conjugated donkey anti- rabbit IgG (1:500; Thermo Fisher Scientific) / Alexa488-conjugated donkey anti- mouse IgG (1:500; Thermo Fisher Scientific) |
| BDA/PV | NA / mouse anti-PV (1:500; Millipore) | Alexa594-conjugated avidin (1:1000, Vector  Laboratories) / Alexa488-conjugated donkey anti- mouse IgG (1:500; Thermo Fisher Scientific) |
